# Dynamic cellular and molecular modulations of diabetes mediated head and neck carcinogenesis

**DOI:** 10.18632/oncotarget.4922

**Published:** 2015-08-21

**Authors:** Chung-Ji Liu, Wan-Jung Chang, Chang-Yi Chen, Fang-Ju Sun, Hui-Wen Cheng, Tsai-Ying Chen, Shu-Chun Lin, Wan-Chun Li

**Affiliations:** ^1^ Department of Oral and Maxillofacial Surgery, MacKay Memorial Hospital, Taipei, Taiwan; ^2^ Department of Medical Research, MacKay Memorial Hospital, Taipei, Taiwan; ^3^ Mackay College of Medicine, Nursing and Management, Taipei, Taiwan; ^4^ Institute of Oral Biology and Department of Dentistry, School of Dentistry, National Yang-Ming University, Taipei, Taiwan; ^5^ Department of Stomatology, Taipei Veterans General Hospital, Taipei, Taiwan

**Keywords:** cell malignancy, diabetes mellitus, head and neck cancer, lymph node metastasis, prognosis

## Abstract

Head and neck squamous cell carcinoma (HNSCC) is one of the most prevalent neoplasms worldwide. While numerous potent dietary insults were considered as oncogenic players for HNSCC development, the impact of metabolic imbalance was less emphasized during HNSCC carcinogenesis. Previous preclinical and epidemiological investigations showed that DM could possibly be correlated with greater incidence and poorer prognosis in HNSCC patients; however, the outcomes from different groups are contradictive and underlying mechanisms remains elusive. In the present study, the changes of cellular malignancy in response to prolonged glucose incubation in HNSCC cells were examined. The results demonstrated that hyperglycemia enhanced HNSCC cell malignancy over time through suppression of cell differentiation, promotion of cell motility, increased resistance to cisplatin, and up-regulation of the nutrient-sensing Akt/AMPK-mTORC1 pathway. Further analysis showed that a more aggressive tongue neoplastic progression was found under DM conditions compared to non-DM state whereas DM pathology led to a higher percentage of cervical lymph node metastasis and poorer prognosis in HNSCC patients. Taken together, the present study confirms that hyperglycemia and DM could enhance HNSCC malignancy and the outcomes are of great benefit in providing better anti-cancer treatment strategy for DM patients with HNSCC.

## INTRODUCTION

While glucose is a major source for cellular energy and bio-molecules, it has long been found that overdosed glucose incubation had deleterious impacts for non-neoplastic cells and could lead to a numbers of adverse complications clinically [1]. In contrast, most cancerous cells are highly addicted to glucose and it is suspected that prolonged high-glucose environment might otherwise provide growth advantage to adopt harsh environment for neoplastic cells [2–4]. Previous study found that high glucose concentration induced gene mutations in a human lymphoblastoid cell line revealing an important underlying mechanism for hyperglycemia-mediated carcinogenesis [5]. Another study showed that hyperglycemia enhanced invasive and migratory activity in pancreatic cancer cells via the oxidative stress mediated stimulation of urokinase plasminogen activator (uPA) further confirmed that an increased glucose level up-regulated malignant phenotypes [6]. Clinically, diabetes mellitus (DM) and neoplasms are both top ranking global healthcare issues affecting millions people [7]. It has long been known that DM and cancer are more frequently diagnosed within the same individuals [8]; however, due to the complex pathogenesis of both disease, it is still difficult to clearly differentiate the interplays between them [9]. More recently, DM has been considered as a potential risk factor, mainly based on the epidemiologic studies, for many neoplasms including head and neck, liver, pancreatic, endometrium, colon/rectum, breast, ovarian and bladder cancers while it may otherwise negatively correlated to prostate cancer [10–14]. Several lines of evidence indicated that DM pathophysiology significantly increased mortality of cancer patients suggesting that DM could also serve as a potential prognostic risk factor for cancer [15–17]. Nevertheless, there is still a lack of experimental data to evaluate dynamic changes and underlying cellular and molecular mechanisms of hyperglycemia mediated tumor development and DM related cancer mortality.

Head and Neck Squamous Cell Carcinoma (HNSCC) is referred to as carcinomas arising from the epithelium lining the sinonasal tract, oral cavity, pharynx and larynx and has become one of the most rapid-growing and poor prognostic neoplasms worldwide [18, 19]. While various factors including alcohol consumption, smoking, areca nut chewing and infection of oncogenic human papillomavirus type 16 (HPV16) could trigger aberrant genetic accumulation, defective epigenetic regulation and tumorous microenvironment resulting in pathological transformation during head and neck carcinogenesis [20–23], the association between imbalanced metabolism, such as DM, and HNSCC development is less emphasized. A retrospective analysis for more than 600 patients with oral squamous cell carcinoma (OSCC) and 574 control cases demonstrated that DM is more frequently detected in the oral cancer group than in the control group (24.3% vs 11.1%) [24]. More recently, it was found that DM patients with OSCC tend to have poorer prognosis based on the detection of a lower overall survival, recurrence-free survival and cancer-specific survival rates compared with non-DM patients [16]. On the contrary, DM duration was not significantly associated with oral cancer prevalence using multivariable adjustment in another study as the statistical analysis using the Taiwanese National Health Insurance database found that use of glucose lowering agent pioglitazone has no noticeable association with oral cancer after adjustment for potential confounders [25]. The null link was also reported in a Barcelona case-control cohort showing none of tested DM treatments (insulin glargine, metformin, sulfonylureas, repaglinide, thiazolidinediones, dipeptidyl peptidase-4 inhibitors and alpha glucosidase inhibitors) influenced oral cancer risk [26]. Interestingly, a recent analysis of over 4.5-million US veterans showed a significantly lower percentage of oral cancer in DM patients implying a negative correlation between DM and oral carcinogenesis [27]. These inconsistent findings from various investigations could possibly result from the differential genetic background of the study populations.

The inconsistent results from former studies might also result from a lack of adjustment for identical aging and dietary associated confounders (obesity, smoking, alcohol consumption, betel nut chewing and hormones) and inaccessibility of biochemical parameters (blood glucose level and hemoglobin A1C) during analysis which lead to a difficulty in precisely correlating DM pathology with HNSCC development. Additionally, to the best of our knowledge, no study has yet reported temporally defined hyperglycemia-mediated regulations during head and neck carcinogenesis. To this end, the current study aims to dynamically examine cellular and molecular modulations in response to differential glycemic treatment using HNSCC cells *in vitro* and to determine the progression of oral cancerous lesions in diabetic mice *in vivo*. Clinically, the influence of DM on the overall survival rates and cancer-specific survival rates of HNSCC patients with or without DM was also retrospectively analyzed. The results demonstrated that prolonged hyperglycemic incubation facilitated cell malignancy both *in vitro* and *in vivo*. HNSCC patients with DM showed worse survival rates compared with non-DM subjects as the DM population with neck lymph node status exhibited the worst cancer-specific survival rates among groups, suggesting DM physiology plays an important role to synergistically regulate HNSCC metastasis.

## RESULTS

### Differential glucose levels modulated cell growth, differentiation and cisplatin resistance in HNSCCs

**Figure 1:**
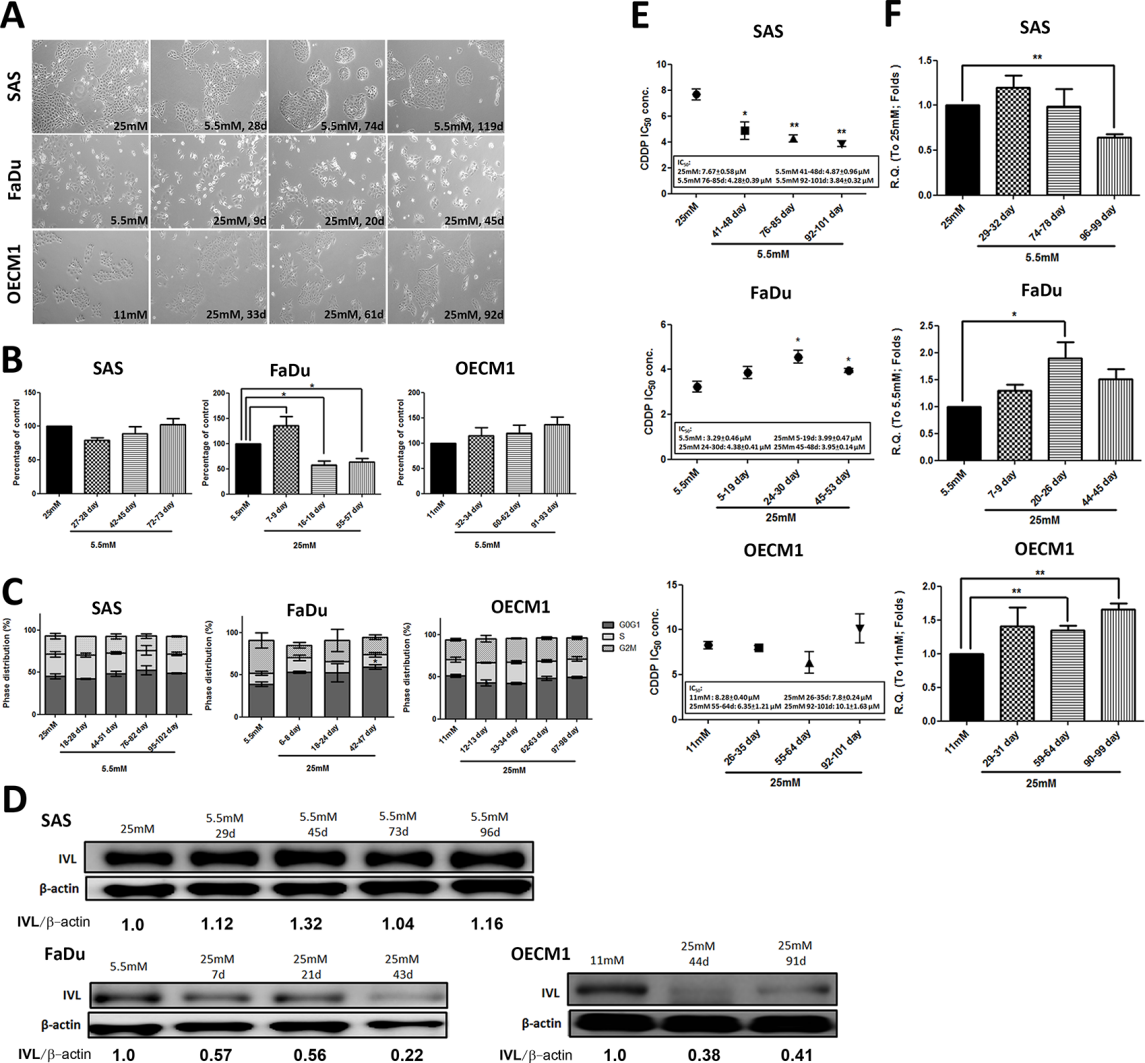
Differential cell growth, decreased cell differentiation and upregulated ABCG2-mediated cisplatin resistance under prolonged high-glucose treatments in HNSCC cells **A.** Glucose switch resulted in cell morphological changes in SAS cells, but not in FaDu and OECM1 cells. SAS cells exhibited less-spiky cell morphology after incubation of prolonged low glucose. Magnification = 200×; Long-term high glucose treatment results in **B.** decreased cell growth using MTT assay and **C.** G0/G1 cell cycle arrest in FaDu cells. There was no significant changes of cell growth and cell cycle distribution in SAS and OECM1 cells in medium containing different glucose levels; **D.** Down-regulated involucrin protein expression was detected under high-glucose environment in HNSCC cells. The involucrin expression was normalized by β-actin protein levels using Image J analysis software; **E.** The significant greater cisplatin IC50 and **F.** increased mRNA expression for the ATP-binding cassette sub-family G member 2 (ABCG2) in HNSCC cells was detected in long-term hyperglycemic cultures. Data are presented as Mean ± SEM (*N* ≥ 3). **p < 0.01; *p < 0.05.

Based on former studies, incubation of insulin-target cells such as podocytes and endothelial cells in 25 mM glucose *in vitro* could result in DM-mediated pathological effects [28, 29]. HNSCC cells SAS (tongue), FaDu (hypopharynx) and OECM1 (oral squamous epithelium) in medium containing 25 mM D-glucose for various periods of time to recapitulate progressive hyperglycemic stimulations were cultivated. There were no significant morphological changes in Fadu and OECM-1 cells in response to glycemic alterations; SAS cells, in contrast, showed clear-edged cell colonies under exposure of lower-glucose environment suggesting SAS cells may become more steady and immobile in hypoglycemic condition (Figure 1A). MTT (Figure 1B) and trypan blue exclusion (Supplementary Figure S1A) assays showed that the changes from physiological to higher glucose concentrations resulted in a distinct reduction in cell growth in FaDu cells. Further examination confirmed that long-term high glucose incubation could result in increased cell apoptosis and significant G2/M cell cycle arrest in FaDu cells, but not in SAS and OECM1 cells (Figure 1C and Supplementary Figure S1B). The cellular variance among SAS, FaDu and OECM1 cells could possibly explained by the distinct glucose uptake capacity, determined by differential intracellular 2-NBDG intake and mRNA expression for glucose transporters (Gluts), in different HNSCC cells (Supplementary Figure S2).

In addition to deregulated cell growth, loss of cell differentiation is also one of the hallmarks during head and neck carcinogenesis as differentiation grading of HNSCC tissues serves as a prognostic indicator clinically [30, 31]. In molecular basis, the specified keratins and epithelial cell-cell interacting proteins serve as differentiation markers [32]. Among them, involucrin was expressed in the granular and upper spinous layers and absent in the basal layer of normal oral mucosa [30]. Papillomas exhibited regular involucrin expression - similar to that in normal squamous epithelium while squamous cell carcinomas showed an irregular distribution of involucrin [33]. The differentiation, based on the involucrin expression, of HNSCC cells under environments with different glucose concentrations was examined to determine glycemia-mediated regulation for cellular differentiation. Despite different cell growth patterns in response to glycemic changes in HNSCC cells, decreased involucrin protein expression was detected in HNSCC cells incubated in high-glucose medium in a time-course manner implying that hyperglycemia progressively impaired cell differentiation (Figure 1D).

HNSCC patients undergoing surgical resection of tumor lesions are often adjuvantly treated with radiation and/or chemotherapy clinically; most patients, however, show loco-regional relapse within five years leading to poor post-surgical outcomes [34]. Recent studies reported that a stem-like HNSCC cell population, referred to as cancer initiating cells (HNSCC-CICs), and ATP-binding cassette (ABC) protein-mediated drug efflux in HNSCC cells might be key molecular regulators for drug sensitivity [35, 36]. To further examine whether high-glucose treatment alters drug sensitivity in HNSCC cells, half maximal inhibitory concentrations (IC50) of cisplatin (CDDP), one of the most commonly used platinum-containing chemotherapeutic drugs, of HNSCC cells incubated in different glycemic environments were determined. Higher IC50 levels were detected in HNSCC cells treated with prolonged hyperglycemia suggesting that high glucose inputs could protect HNSCC cells from cisplatin-mediated cytotoxicity (Figure 1E), probably via significant increased expression of the drug-resistant mediator ABCG2 mRNA (Figure 1F). Interestingly, there are no significant changes in CIC populations determined by aldehyde dehydrogenase activity (ALDH) [37] and Oct4 mRNA expression in HNSCC cells cultured in differential glucose environments (Supplementary Figure S2) indicating hyperglycemia facilitated cisplatin resistance in whole HNSCC cells rather than solely in HNSCC-CICs.

### Elevated glucose level promotes cell motility via epithelial-mesenchymal transition and cytoskeletal rearrangement

**Figure 2:**
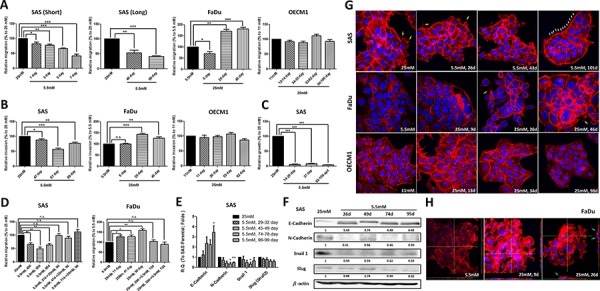
Hyperglycemia increased cell motility via up-regulated epithelial-mesenchymal transition (EMT) and F-actin rearrangement Transwell-based **A.** migration, **B.** invasion assay and **C.** anchorage-independent growth showed greater cell motility in SAS and FaDu cells, but not OECM1 cells, in response to prolonged hyperglycemic cultures compared with cells incubated in low-glucose environments; **D.** Cell migration was modulated after switching back to parental medium in SAS and FaDu cells; **E.** Real-time RT-PCR and **F.** Western blot analysis for EMT markers showed up-regulated E-cadherin and suppressed N-cadherin and Snail in SAS cells cultured with low-glucose medium over time. The relative expression of EMT proteins was normalized with β-actin protein levels using Image J analysis software; **G.** Filopodia-like (yellow arrows) and lamellipodia-like (white arrows) F-actin were detected in SAS and FaDu cells in hyperglycemic condition and low-glucose medium, respectively; **H.** The enriched F-actin expression and vertical multi-cellular stack indicated by green arrows was detected in FaDu cells under high-glucose culture. Data are presented as Mean ± SEM (*N* ≥ 3). ***p < 0.001; **p < 0.01; *p < 0.05; n.s.= non-significant.

Being mobile is a key step for cancer cell metastasis. Using a transwell-based assay, cell migration and invasion as well as soft-agar mediated anchorage-independent growth of HNSCC cells cultured in different glucose levels were analyzed. The results showed that SAS cells exhibited decreased migration under 1-week incubation in lower-glucose conditions (Figure 2A) and migration and invasion activity remained minimal in prolonged low-glucose cultures (up to 99 days) (Figure 2A and 2B). Further analysis for anchorage-independent growth for SAS cells cultured in lower-glucose medium demonstrated that less environmental glucose led to slower growth of SAS cells on soft-agar (Figure 2C). In contrast, FaDu cells showed significantly greater cell motility in response to high glucose while external glucose is insufficient to alter cell migration/invasion of OECM1 cells after long-term culture (Figure 2A and 2B). Interestingly, the glycemia-mediated changes for cell migration in SAS and FaDu cells could be rapidly attenuated when the cells were switched back into parental conditions (Figure 2D) indicating that regulation for HNSCC cell motility by extrinsic glucose input is highly dynamic and reversible.

For most carcinomas, distant invasion is often initiated by loss of epithelial intercellular contacts and a shift towards a mesenchymal phenotype, referred to as epithelial mesenchymal Transition (EMT), exacerbates motility and invasiveness of many cell types and is often considered a prerequisite for tumor infiltration and metastasis [38]. In addition, recent studies also demonstrated that EMT is essential for metastatic phenotype during HNSCC progression [39]. Quantitative RT-PCR and Western blot analysis showed increased E-cadherin expression and reduced N-cadherin and Snail levels predominantly in SAS cells, but not in FaDu and OECM1 cells, cultivated in a lower-glucose medium providing a potential mechanistic cue of glucose-mediated regulation for HNSCC cell motility (Figure 2E, 2F and Supplementary Figure S3A).

Cancer metastasis is a multi-stage process involving in invasion into surrounding tissue, intravasation, migration into blood or lymph circulating systems, extravasation and growth at a new site [40]. During metastasis, cell motility could be driven by dynamic polymerization of actin molecules into polarized filaments, termed F-actin [41]. In molecular basis, Formin homology (FH) molecule and Enabled/Vasodilator-stimulated phsphoproteins (Ena/VASP) could promote the generation of spiky filopodia and Actin related proteins 2 and 3 (Arp2/3) protein regulated arc-like sheets of F-actin leading to lamellipodia formation [42]. It was shown that the cellular F-actin level was positively correlated with bladder cancer risk clinically implying up-regulated F-actin expression and structural organization might be one of the indicators of tumor malignancy [43]. To further correlate the cytoskeletal rearrangement with glycemia-mediated cellular motility, the fluorescently conjugated phalloidin was used to visualize expression and organization of F-actin in HNSCC cells treated with high/low glucose concentrations. While F-actin expression is not significantly different in SAS cells cultured in different glycemic conditions, the obvious change from filopodia-enriched cells (in 25 mM glucose) into lamellipodia-edged cells (in 5.5 mM glucose) was detected (Figure 2G, upper row). In contrast, the dramatically increased F-actin expression and enriched filopodia-like protrusions were found in FaDu cells under hyperglycemic cultures (Figure 2G, middle row) suggesting that cytoskeletal rearrangement could be another underlying mechanism for differential cell migratory activity in response to glycemic stimulation. Strikingly, 3-dimensional Confocal microscopic analysis showed vertical multi-cellular stacks in FaDu cells cultured in long-term hyperglycemic condition, implying that extrinsic high-glucose challenge could trigger multi-directional migration for FaDu cells, but not for SAS or OECM1 cells (Figure 2H and Supplementary Figure S3B). As tumor cell aggregation is a key component of the metastatic process in either seed-and-soil hypothesis or mechanical trapping theory [44], the unique pattern of FaDu cells in response to high-glucose cultures confirmed that enhanced F-actin could be essential supporting greater cell-cell contact to form cell aggregates, like metastatic tumors *in vivo*.

### Multiple molecular mechanisms involved in glycemia-mediated promotion for HNSCC malignancy

**Figure 3:**
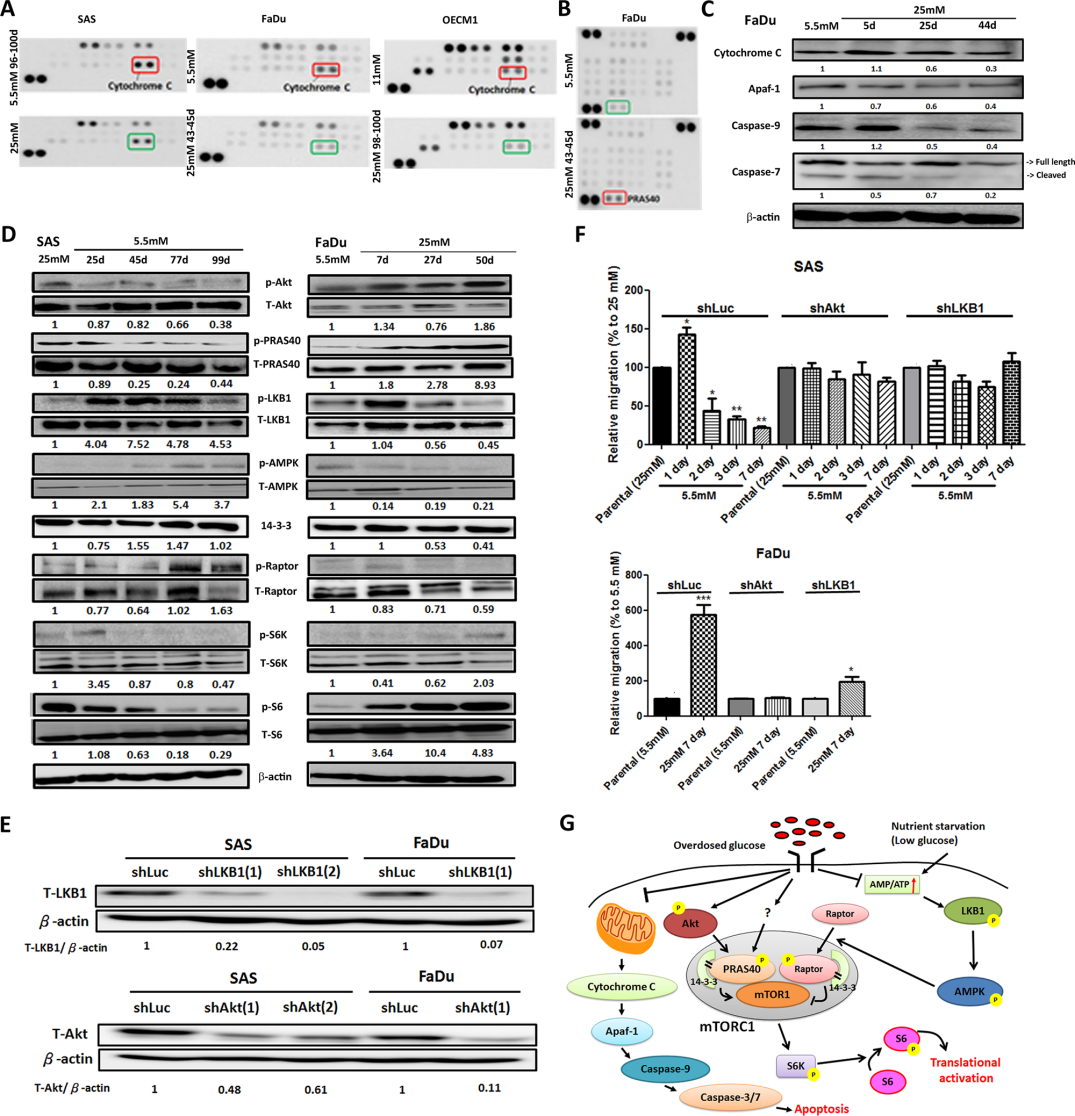
Molecular regulation of hyperglycemia-mediated carcinogenic promotion in head and neck cancer cells **A.** Mitochondrial redox sensor Cytochrome c is down-regulated in high-glucose culture condition in HNSCC cells using Human Cell Stress Antibody Array; **B.** Up-regulated expression of AKT/PKB substrate phospho-PRAS40 (T246) was shown in high-glucose treated FaDu cells using Human Phospho-Kinase Array; **C.** Western blot analysis showed Cytochrome c and its downstream molecules Apaf-1, caspase 9 and caspase 7 are down-regulated in FaDu cells cultured in high-glucose medium. The relative expression Cytochrome c, Apaf1, caspase 9 and cleaved caspase 7 was normalized with β-actin protein levels using Image J analysis software; **D.** Western blot analysis for molecules in Akt/AMPK-mTORC pathway showed differential expression in SAS and FaDu cells under various glycemic environments. The expression of active Akt, PRAS40 and mTORC1 downstream regulator p70S6K and its direct target S6 proteins were upregulated under high-glucose treatment while the increased active LKB1, AMPK and Raptor proteins were detected in SAS and FaDu cells treated with prolonged low-glucose condition. The activity of different proteins was defined as the ratios of phosphorylated protein expression to total protein levels using Image J analysis software; **E.** Western blot analysis showed successful establishment of Akt and LKB1 deficient SAS and FaDu cells using shRNA-mediated method. The knockdown efficiency was determined by Akt/LKB1 to β-actin protein expression ratio using Image J analysis software; **F.** Glycemia-mediated migration activity is abolished in Akt and LKB1 deficient SAS and FaDu cells. Data are presented as Mean ± SEM (*N* ≥ 3). ***p < 0.001; **p < 0.01; *p < 0.05; **G.** Summary of glycemia-mediated molecular regulations in HNSCC cells.

To further elucidate glycemia-mediated regulatory players for HNSCC malignancy, proteomic analysis of HNSCC cells cultured in low- and high-glucose medium using large throughput antibody arrays were performed (Figure 3A, Figure 3B and Supplementary Figure S4). The results showed that heat shock protein 70 (HSP 70), A disintegrin and metalloprotease with thrombospondin motifs 1 (ADAMTS1), Beta-catenin as well as phosphorylated proline-rich Akt substrate of 40 kDa (PRAS40), WNK Lysine-deficient protein kinase 1 (WNK1) and Signal transducer and activator of transcription 3 (STAT3) were up-regulated in high-glucose treated HNSCC cells. In contrast, higher expression of mitochondrial apoptosis mediator Cytochrome c and cell cycle regulator phosphorylated p53 proteins were found in HNSCC cells cultured under lower glucose conditions.

The Cytochrome c mediated apoptosis pathway was further emphasized. Cytochrome c resides in the mitochondrial intermembrane serving as a redox carrier for electric transport chain [45]. Previous studies demonstrated that release of Cytochrome c could trigger downstream caspase activity resulting in formation of apoptosomes leading to subsequent cell death in HNSCC both *in vitro* and *ex vivo* [46]. Western blot analysis showed that major components involving in Cytochrome c mediated apoptotic pathways including Cytochrome c, Apaf-1, Caspase 9 and Caspase 3/7 were down-regulated in FaDu cells (Figure 3C) and SAS cells (Supplementary Figure S5A) cultivated in high-glucose environment suggesting long-term hyperglycemia protected HNSCC cells from Cytochrome c mediated cytotoxicity. The Akt/AMPK-mTORC1 pathway, one of the common molecular pathways underlying HNSCC pathogenesis, was also examined [47]. It was shown that aberrant overactivation of epidermal growth factor receptor or insulin-like growth factor receptor signals trigger phosphatidylinositol 3-kinase (PI3K)/Akt protein kinases leading to a sequential phosphorylation of PRAS40 that in turn binds to 14-3-3 protein in cancer cells. The complex could then facilitate mTORC1 activity and modulate downstream ribosomal protein S6 (S6) and eIF4E-binding protein 1 (4E-BP1) proteins thereby resulting in up-regulated cell growth [48]. On the other hand, nutrition deprivation activates 5′ AMP-activated protein kinase (AMPK) phosphorylation by liver kinase B1 (LKB1) and active AMPK phosphorylates Raptor protein stimulating 14-3-3 association leading to suppressed mTORC1 activity [49]. Taken together, nutrient inputs could regulate the Akt/AMPK-mTORC1 pathway that would possibly contribute to glycemia-mediated cancer malignancy. Western blot analysis for proteins involving in Akt/AMPK-mTORC1 pathway in HNSCC cells incubated in different glucose showed that high-glucose environment activated Akt, PRAS40 but inhibited LKB, AMPK1 and Raptor proteins (Figure 3D). The combined Akt- and AMPK-dependent regulations up-regulated phosphorylated S6 expression promoting protein translation to support cell growth (Figure 3D and Supplementary Figure S5B). To further validate the glycemic regulations of Akt/AMPK-mTORC1 pathways, the changes of migratory activity of shRNA-mediated Akt and LKB1 deficient SAS and FaDu cells (Figure 3E and Supplementary Figure S7) under low- and high-glucose culture conditions were examined. While there are no significant morphological and growth difference for Akt/LKB1 deficient cells compared with shLuc control cells (Supplementary Figure S8), glycemia-mediated cell migration was abolished in Akt/LKB1 deficient SAS and FaDu cells in response to glycemic changes (Figure 3F) supporting the significance of Akt/AMPK-mTORC1 pathways in regulating glycemia-mediated head and neck tumorigenesis. In summary, the Cytochrome c mediated apoptosis and Akt/AMPK-mTORC1 pathway play important roles in controlling cellular malignancy in HNSCC cells under prolonged treatment of differential glucose levels (Figure 3G).

### Hyperglycemia/diabetic condition facilitated 4-NQO induced tongue neoplastic progression via activation of mTORC1 effector

**Figure 4:**
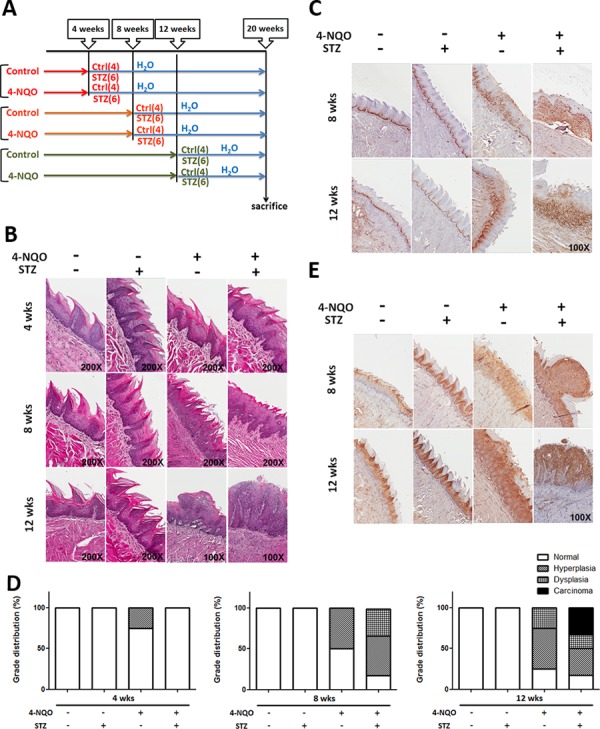
Diabetic conditions promoted oral carcinogenesis *in vivo* **A.** Experimental scheme to establish DM mice with 4NQO induced oral cancer. All animals were sacrificed after 20 weeks of initial 4-NQO feeding. The number of animals for each experimental group was shown in parenthesis. Histological analysis for **B.** Hematoxylin & Eosin, **C.** Ki67 and **E.** mTORC1 downstream regulator phosphorylated S6 protein showed that the more aggressive oncogenic lesions were detected in DM mice compared with time-matched non-DM animals; **D.** Quantification of precancerous/tumor incidence in mice treated with STZ and 4-NQO.

Recent studies found that DM could activate the erbB2/B3-Ras/Raf/MAPK signaling pathway which leads to increased cell proliferation in oral tumors using a 4-Nitroquinoline 1-oxide (4-NQO) treated rat model [50]; nevertheless, it remains unknown whether cell growth and molecular cues of early precancerous lesions could also be modulated under hyperglycemic/diabetic conditions. It was previously shown that the neoplastic tumors could be detected under treatment of 50–100 μg/ml 4-NQO in drinking water for 20–26 weeks [51], in order to induce early cancerous lesions, 4-,8- and 12-week 4-NQO treatments to stimulate precancerous oral lesions was first applied followed by streptozotocin (STZ) injection to elicit DM (Figure 4A). Histological analysis, based on H&E staining and Ki67 expression, confirmed that hyperplastic/dysplastic tongue epithelium could be detected after 8-week and 12-week treatments of 50 μg/ml 4NQO indicating effective 4-NQO induced transformation of oral epithelium (Figure 4B, 4C). The insulin-producing pancreatic islet beta cells were destroyed and elevated blood glucose levels were detected within 2 weeks after 5-day consecutive low-dose (50 mg/kg) STZ injection and remained hyperglycemia until sacrifice (Supplementary Figure S6). Histological analysis for tissue sections from mouse tongues under different treatments showed that a greater percentage of progressive tongue tumor lesions were detected in 8- and 12-week treated DM mice compared with non-DM groups suggesting DM physiology facilitated 4-NQO-induced tumorigenesis (Figure 4D). Interestingly, the enriched expression of mTORC1 downstream effector phospho-S6 protein was detected in 4-NQO induced tongue neoplastic lesions further confirming the oncogenic role of mTORC1 associated components (Figure 4E).

### Diabetes is associated with risk of mortality and cancer-specific survival rates in patients with HNSCC

**Figure 5:**
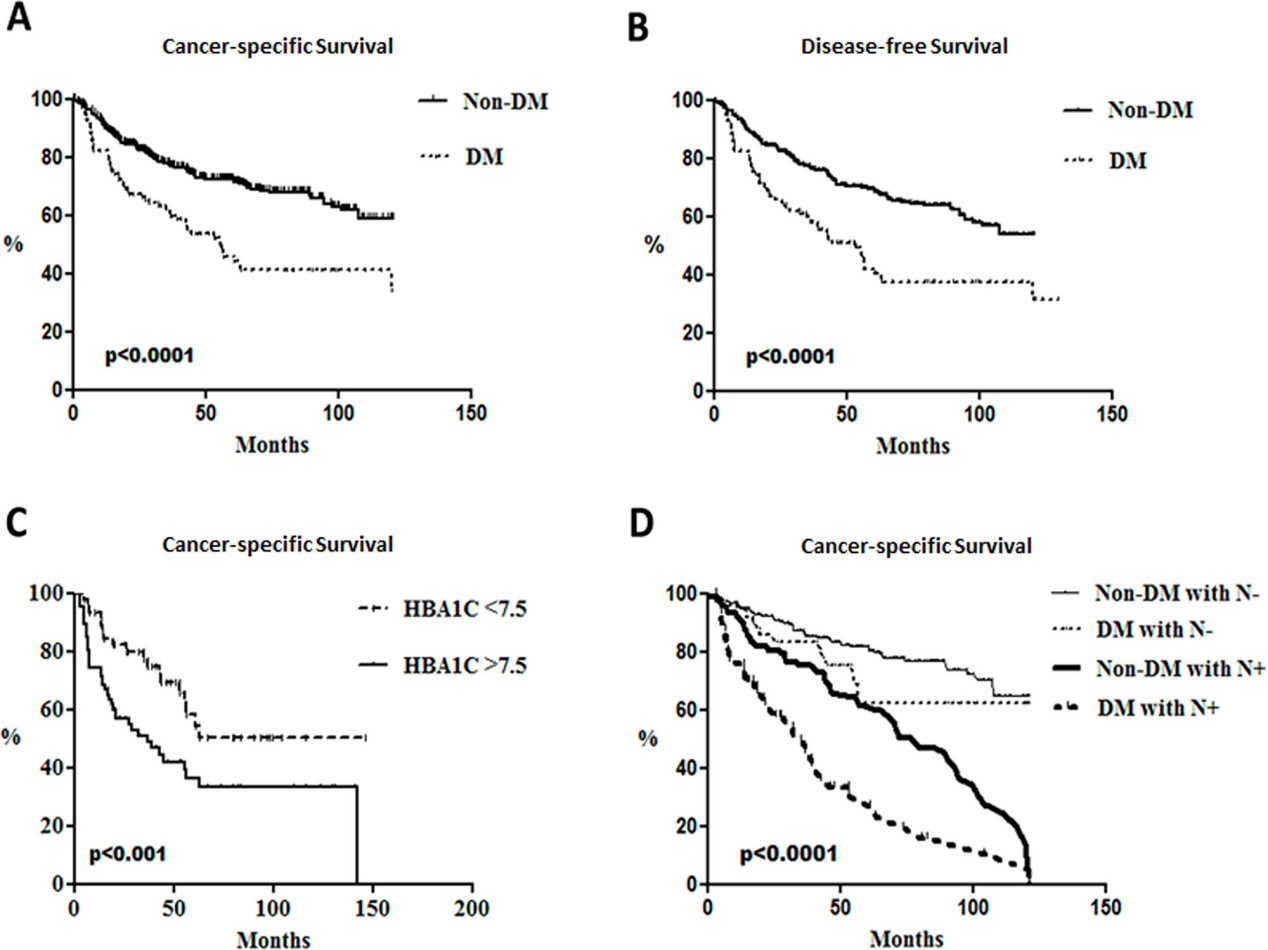
Poorer survival rates in head and neck cancer patients with diabetes and lymph node metastasis Kaplan-Meier survival analysis for **A.** cancer-specific survival rates and **B.** disease free survival rates between DM (*N* = 126) and non-DM subjects (*N* = 487) (*p* < 0.0001) and cumulative cancer-specific survival rates of **C.** DM patients with differential HbA1c status (*p* < 0.001) and **D.** groups associated with neck lymph node status in DM patients and non-DM subjects (*p* < 0.0001).

Potential clinic significance of DM on patients with HNSCC was retrospectively examined. The database with 613 HNSCC patients containing 126 DM patients (115 males and 11 females) and 487 non-DM subjects (454 males and 33 females) were analyzed. Different clinical factors including gender, smoking and drinking habits, median follow-up time, tumor size, tumor site, cell differentiation and perineural invasion were all similar between DM and non-DM groups (Supplementary Table S2). By using univariant analysis, cervical lymph node status, tumor stage, perivascular permeation and survival status were different between DM and non-DM groups as the multivariate logistical regression analysis showed that age, survival status and lymph node status were associated with DM (Table 1A). While our data also revealed a hazard ratio of 2.89 for overall mortality in DM patients (Supplementary Table S3), DM may be an independent factor for cervical lymph node metastasis, the most important clinical factor for the cancer-specific survival rates (CSS) of HNSCC patients (Table 1A). Kaplan-Meier survival analysis indicated that DM patients had worse CSS (Figure 5A) and disease-free survival rates (DFS, Figure 5B). Interestingly, a cut-off value of 7.5% of pre-operative hemoglobin A1C (HbA1C) could also be a significant determinant for CSS in this cohort (Figure 5C). Further analysis for potential confounding factors for DM-mediated mortality in HNSCC patients was also performed and the results using multivariate logistical regression analysis indicated that only lymph node status is related to DM-related CSS (Table 1B); whether lymph node status acts as an independent risk factor for DM-associated CSS was therefore examined. All participants were classified into 4 groups based on DM and nodal status and Cox regression models showed no significant difference between lymph node negative patients with or without DM while DM patients with lymph nodes status exhibited the worst CSS implying that DM and lymph node status could synergistically regulate HNSCC prognosis (Figure 5D).

Table 1DM-associated Factors for HNSCC Prognosis

**Table:** **A.** Univariant analysis for tumor onset age, smoking and drinking habits, cervical lymph node status, cancer stage, lymphovascular permeation and survival status were different between DM and non-DM groups. Multivariate logistical regression analysis showed age, survival status and lymph node status were associated with DM.

	Non-DM (*N* = 487)		DM (*N* = 126)		Total (*N* = 613)		*P*-value	OR	95% C.I.	*P*-value
	*N*	%	*N*	%	*N*	%				
**Gender**							0.449			
Male	454	93.2%	115	91.3%	569	92.8%				
Female	33	6.8%	11	8.7%	44	7.2%				
**Tobacco smoking**							0.4			
Daily	290	59.5%	70	55.6%	360	58.7%				
Non-smoker	197	40.5%	56	44.4%	253	41.3%				
**Alcohol abuse**							0.6			
Daily	51	10.5%	15	11.9%	66	10.8%				
None or social	436	89.5%	111	88.1%	547	89.2%				
**Age** (yr)	52.46 ± 11.30		56.42 ± 9.46		53.28 ± 11.06		*p* < 0.001	1.04	1.02–1.06	
**Survival status**							*p* < 0.001			
Dead	128	26.3%	70	55.6%	198	32.3%		2.59	1.65–4.05	*p* < 0.001
Survival	359	73.7%	56	44.4%	415	67.7%		1.00		
***N* status**							*p* < 0.001			
*N* = 0	317	65.4%	55	43.7%	372	60.9%		1.00		
*N* > 0	168	34.6%	71	56.3%	239	39.1%		1.68	1.01–2.79	0.044
**Tumor size**							0.081			
T1 – T3	215	44.3%	45	35.7%	260	42.6%				
T4	270	55.7%	81	64.3%	351	57.4%				
**Tumor stage**							*p* < 0.001			
I–II	126	26.0%	14	11.1%	140	22.9%		1.00		
III–IV	359	74.0%	112	88.9%	471	77.1%		1.55	0.8–3.03	0.196
**Lymphovascular permeation**							0.031			
Positive	81	16.9%	31	25.4%	112	18.6%		0.89	0.51–1.54	0.669
Negative	398	83.1%	91	74.6%	489	81.4%		1.00		
**Cell differentation**							0.572			
Well	178	36.6%	42	33.3%	220	35.9%				
Moderate	278	57.1%	73	57.9%	351	57.3%				
Poor	31	6.4%	11	8.7%	42	6.9%				
**Perineural invasion**							0.306			
Negative	381	78.9%	91	74.6%	472	78.0%				
Positive	102	21.1%	31	25.4%	133	22.0%

**Table:** **B.** Multivariate logistical regression analysis determined the risk factors associated with mortality in DM and non-DM subjects.

	DM			Non-DM		
	OR	95% C.I.	*P*-value	OR	95% C.I.	*P*-value
***N* status**						
*N* = 0	1.00			1.00		
*N* > 0	4.19	1.67–10.53	0.002	1.76	1.02–3.04	0.042
**Tumor size**						
T1–T3	1.00			1.00		
T4	2.22	0.81–6.1	0.12	3.40	1.79–6.45	*p* < 0.001
**Stage**						
I–II	1.00			1.00		
III–IV	1.10	0.2–5.98	0.911	1.60	0.61–4.17	0.337
**Lymphovascular permeation**						
Positive	1.74	0.62–4.93	0.295	1.36	0.72–2.58	0.339
Negative	1.00			1.00		
**Cell differentation**						
Well	1.00			1.00		
Moderate	1.22	0.5–2.97	0.666	1.96	1.18–3.26	0.009
Poor	2.94	0.48–18.03	0.243	2.39	0.97–5.88	0.057
**Perineural invasion**						
Negative	1.00			1.00		
Positive	0.82	0.3–2.2	0.69	1.53	0.89–2.62	0.123

## DISCUSSION

Numbers of preclinical and epidemiological studies showed that DM could possibly be correlated with greater incidence and poorer prognosis in patients with HNSCC; nevertheless, the outcomes are contradictive and underlying mechanisms remains elusive. In the present study, the dynamic regulations for cellular malignancy in HNSCC cells and the impact of DM on head and neck carcinogenesis were examined. The results demonstrated that overdosed glucose promoted HNSCC malignancy in time-course manner via various cellular and molecular mechanisms including modulation of cell growth, inhibition of cell differentiation, enhanced cell motility, increased drug resistance and upregulation of nutrient-mediated oncogenic pathways. Moreover, the detection of the more aggressive 4-NQO induced tongue neoplastic lesions in DM mice and worsened HNSCC prognosis in DM patients further confirmed that diabetic pathology facilitated oral tumorigenesis. It is worth noting that prolonged high-glucose incubation had an adverse impact for cell growth in FaDu cells as the remaining cells exhibited greater malignant phenotype and activated anti-apoptotic molecular profile compared to cells cultivated in low-glucose conditions, suggesting that hyperglycemia might provide a growth advantage for more aggressive HNSCC cells over less malignant ones. In addition, our results also indicated that hyperglycemia-mediated promotion for HNSCC cell malignancy is reversible while the hyperglycemia-mediated promotion for cell motility in SAS and FaDu cells was abolished when the cells were switched to a lower-glucose environment. Clinically, it was shown that proper glucose control leads to better clinical outcomes in breast cancer patients [52] highlighting the significance to tightly control blood glucose in DM patients with neoplastic lesions.

The Akt/AMPK-mTORC1 pathway was defined as one of the potential glucose related regulatory signaling cues for HNSCC cell development in the current study. Indeed, numerous studies were reported as showing differential anti-cancer effects of routine DM medication metformin, an AMPK activator, on various cancer types [53, 54]. One of the working mechanisms of anti-cancer impacts of metformin is to specifically target metabolic differences between normal and tumorous physiology. For example, metformin might regulate tumorigenesis through the systemic reduction of growth factor insulin and the induction of energetic stress by oxidative phosphorylation [55]. For HNSCC, metformin administration was effective to modulate cell growth via global protein translational regulation *in vitro* as well as to inhibit mTORC1-mediated cell proliferation in oral epithelial basal cells *in vivo* implying its significance as anti-neoplastic and anti-diabetic medication in DM patients with HNSCC [56, 57]. Interestingly, a very recent study found that metformin facilitated autophagy and triggered apoptosis by down-regulation of oncogenic factor STAT3, one of the glycemia-mediated regulators revealed using the protein array in this study (Supplementary Figure S4B), in esophageal SCC [58] further suggesting potential interplays between different glycemic oncotargets.

In addition to Cytochrome c mediated apoptosis and Akt/AMPK-mTORC pathways, the molecular cues differentially expressed in HNSCC cells under low- and high-glucose environments revealed by human antibody array analysis (Supplementary Figure S4C) could also be important glycemia-mediated malignant determinants. HSP70 is a protein chaperon abundantly expressed in malignant tumors and mainly assists with structural folding of newly synthesized polypeptide, while its expression correlates with increased cell proliferation, poor differentiation, lymph node metastases and poor prognosis [59]. Beta-catenin, a downstream regulator of the Wnt signaling pathway, targets genes regulating cell proliferation and apoptosis thereby mediating cancer initiation and progression. Previous studies showed that high glucose levels could enhance WNT/β-catenin signaling in cancer cells leading to increased proliferation, survival and senescence bypass whereas nuclear beta-catenin expression is correlated with tumor malignancy in oral cancer tissues [60]. ADAMTS1 could bind and degrade extracellular matrix components and plays an important role in triggering pulmonary metastasis of mammary carcinoma [61]. The detection of up-regulated ADAMTS1 expression in high-glucose cultured HNSCC cells could therefore be one of the molecular promoters for hyperglycemia-mediated cell motility. Another hyperglycemia activated phospho-protein, WNK1, could regulate multiple signaling pathways related to cell proliferation, ion channel regulation and protein synthesis [62]. It was shown that phosphorylated WNK1 is involved in the regulation of GLUT1 in noninsulin target cells suggesting its role in controlling Warburg metabolism in cancer cells [63]. Differential phosphorylation of tumor suppressor p53 was detected in OECM1 cells treated with lower glucose. Recent studies have shown that phosphorylation of p53 protein (s15) may play a critical role in the stabilization and functional activation of p53 under cellular stress. The p53 phosphorylation by the DNA-dependent protein kinase (DNA-PK) *in vitro* led to reduced binding to its negative regulator, MDM2, and undergoes G1 arrest in response to DNA damage [64]. In summary, the protein array analysis uncovered candidate glycemia-mediated regulatory signals in a larger-scale manner, as future validation for candidate molecular pathways is required.

While the clinical analysis and the present finding showed the impact of DM as a strong prognostic factor for survival of HNSCC patients after curative surgical intervention [16, 24], the DM-mediated modulations for HNSCC development were also evident in a number of previous studies. Early investigations found that the Oral Leukoplakia (OL) prevalence was higher in DM patients than in non-DM subjects (6.2% vs 2.2%) [65]. A larger cohort, The Study of Health in Pomerania (SHIP), also showed greater Hemoglobin A1C (HbA1C) level in OL subjects revealing the potential correlation of DM and OL prevalence [66]. In contrast, a cohort study analyzing population with oral cancer in Kerala, India, showed no significant association between DM and premalignant lesion Oral Submucous Fibrosis (OSF) in men but a correlation was detected among women suggesting a sex-dependent manner for DM-mediated regulation of early head and neck neoplasm development [67]. Several DM associated clinicopathological parameters were also reported to correlate survival rates in HNSCC subjects [68]. For example, A study from The Netherlands analyzing the 2-year, 5-year survival and age-standardized incidence of HNSCC in the Dutch population from 1989 to 2011 showed that incidence of oral, oropharyngeal and hypopharyngeal squamous cell carcinoma has increased and survival rates was improved whereas the laryngeal carcinoma incidence changes vary in gender and the survival rate remained unchanged [69]. Another study reported that the secondary primary malignancy occurred in patients with laryngeal and hypopharyngeal squamous cell carcinoma but exhibited different pattern and survival rates indicating potential site-specific HNSCC prognosis [70]. The site-specific variant HNSCC prognosis was examined in our database and the result showed no significant difference among patients with different disease sites and their survival status (unpublished data). In the present cohort, a number of variables including age, tumor stage, tumor size, cell differentiation state, vascular permeation and cervical lymph nodal status were adjusted to eliminate potential bias in exploring independent determinants for DM-mediated HNSCC development. Among all candidate predictors, the lymph node status is the only factor associated with disease prognosis indicating that DM may affect the metastasis to some extent. Indeed, *in vitro* results did show significantly greater cell motility in HNSCC cells cultured under prolonged high-glucose environments supporting the theory that DM could be a risk factor for HNSCC progression and cervical lymph node metastasis. Nevertheless, it is worth noting that the *in vitro* data are not completely corresponding to *in vivo* or clinical observations in the present study suggesting that, in contrast to hyperglycemia-mediated malignancy changes, the underlying mechanisms for DM-associated tumorigenesis or poor prognosis in patients with HNSCCs would likely to be more complicated.

In summary, we concluded that long-term cultivation under high-glucose environments promoted HNSCC malignancy both *in vitro* and *in vivo*. Clinical analysis further supported that DM pathogenesis could be an independent factor for worsening lymph nodal metastasis and poorer patient survival. The overall outcomes are not only beneficial in understanding how hyperglycaemic insult regulates head and neck carcinogenesis but also in supporting the requirement of tight glycemic control for clinical practice to achieve better prognostic outcomes for DM patients with neoplasms.

## MATERIALS AND METHODS

### General reagents

Primers for RT-PCR and antibodies for immunostaining and Western blot analysis were summarized in Supplementary Table S1.

### Cell cultures and clinical information

Culture conditions for human HNSCC OECM1, SAS and FaDu cells were previously described [71–73]. The clinical study was approved by the Institutional Review Boards of MacKay Memorial Hospital, Taiwan.

### Establishment of Akt/LKB1 deficient HNSCC cells

The plasmids encoding small hairpin RNA (shRNA) targeting Akt/LKB1 genes purchased from National RNAi Core Facility (NRCF), Academic Sinica, Taiwan, were amplified following the standard protocol provided NRCF. The lentiviral vectors containing shAkt/shLKB1 and the control shRNA targeting Luciferase (shLuc) or beta-galactosidase (shLacZ), were generated in 293T cells. Akt and LKB1 deficient SAS and FaDu cells were cultured in medium containing 4 μg/ml puromycin for further experiments.

### 2-NBDG glucose uptake assay

For glucose uptake assays, cells were grown for 24 hrs and 10 mg/ml 2-NBDG (Life Technology) was added to the media for 1 hr and the fluorescence was visualized under a Zeiss Axio Vert A1 fluorescence microscope in the Instrumentation Resource Center in National Yang-Ming University.

### Cell cycle and apoptosis analysis

For cell cycle analysis, 1 × 10^6^ cells were suspended in PBS and fixed in 70% ethanol at −20°C for 1 hr. Fixed cells were re-suspended in propiodium iodide (PI) staining solution for 30 minutes at room temperature. Cell apoptosis/necrosis was determined using Annexin V-FITC Apoptosis Detection Kit (Strong Biotech Corporation). The number of 1 × 10^6^ cells was used for assay. Cells were stained with fluorescently labeled buffer containing 1 μg/ml PI and Annexin V FITC in dark at room temperature for 15 minutes. The cell cycle and apoptosis were analyzed on Beckman Coulter Cytomics FC500 Flow Cytometry at Instrumentation Resource Center, National Yang-Ming University, Taiwan.

### Migration/invasion assay

The transwell based migration/invasion assays were performed to evaluate cell motility. As for migration assay, the number of 5 × 10^4^∼10^5^ cells were seeded onto 8 μm-pore transwell chambers 16 hours before experiment. The cells in upper chamber were incubated in serum-free medium while the medium containing 30% fetal bovine serum (FBS) was applied in lower chamber. To identify migrating cells, the cells on upper side (non-migrating cells) of membrane were removed using Q-tips as the cells attached on the bottom of membranes were fixed in 4% Paraformaldehyde (PFA) and stained with crystal violet for 2 hr. For invasion assay, the similar protocol is applied. The transwell chambers were coated with 50 μg/ml matrigel diluted in cold serum-free medium as for 2.5 μg/ml and the incubation time is for 24∼48 hours. The number of migrating/invading cells from 5–8 different microscopic areas of each membrane was analyzed and the results are presented as Mean ± SEM.

### Anchorage-independent growth assay

Cells were suspended in 1.3% methylcellulose in culture media at a density of 1 × 10^5^ per well and plated on a layer of 0.9% agarose in culture media containing 15% FBS. Cells were then cultured at 37°C incubator for 10 or 14 days. The number of colonies present with diameters >50 μm in more than five fields per well using triplicate experiments was counted and the results are presented as Mean ± SEM.

### Reverse transcription and polymerase chain reactions (RT-PCR)

Total RNA was extracted using TRIzol reagent following the manufacturer's instructions. Purified RNA concentration was measured by NanoDrop TM 1000 spectrophotometer; reverse transcription was carried out using SuperScript III First-Strand Synthesis System (Invitrogen). Quantitative PCR reactions containing cDNA, KAPA SYBR FAST ABI PRISM 2x qPCR Master Mix, sense and antisense primers were processed in StepOnePlus™ Real-Time PCR System (ABI Biosystems) at Instrumentation Resource Center, National Yang-Ming University.

### Western blot analysis

Protein lysates were isolated using RIPA lysis buffer. For protein analysis, equal amount of proteins were denatured by incubation at 95°C for 10 minutes and separated in 10–12.5% SDS-PAGE gel. The proteins were then transferred to PVDF membrane and blocked with 5% non-fat milk in PBS-T for 1 hour at room temperature to reduce background staining. The indicated primary antibody was applied at 4°C for 16–20 hours and the membrane is incubated with horseraddish peroidase (HRP) conjugated secondary antibody in PBS-T with 5% non-fat milk for 1 hour at room temperature. The ECL based system and FUJIFILM Luminescence Imaging System LSA-4000 system at Instrumentation Resource Center, National Yang-Ming University was used to visualize protein signals.

### Cisplatin treatment & MTT assay

HNSCC cells were treated with various concentrations (0 to 40 μM) of cisplatin for 48 hours and the relative viable cells were determined by MTT method. In brief, the drug-treated cells were washed with PBS and incubated with 1 mg/ml 3- (4,5-cimethylthiazol-2-yl)-2,5-diphenyl tetrazolium bromide (MTT) dissolved in the culture medium for 3 hours. After removal of the culture medium, cells were then lysed with DMSO and OD 570 was measured by a microplate reader.

### ALDH assay

Aldehyde dehydrogenase (ALDH) activity was measured using ALDEFLUOR™ Kit (Stemcell technologies). In brief, testing cells were incubated in Aldefluor assay buffer containing reaction substrate, BODIPY-aminoacetaldehyde (BAAA), at 37°C for 30 min. ALDH+ cells catalyzed BAAA to its fluorescent product, BODIPY-aminoacetate (BAA) and analyzed using Beckman Coulter Cytomics FC500 Flow Cytometry at Instrumentation Resource Center, National Yang-Ming University. As for negative control, a specific ALDH inhibitor, Diethylaminobenzaldehyde (DEAB) was used to define background ALDH activity.

### Human antibody array

The Human Cell stress and Phospho-antibody arrays (R&D System Inc.) were used to identify potential molecular pathways in response to glycemic challenge following the manufacturer's procedure. The amount of 600 μg protein lysates were used for each run and signals were analyzed using FUJIFILM Luminescence Imaging System LSA-4000 system at Instrumentation Resource Center, National Yang-Ming University.

### Animal procedure

All animal studies were conducted in accordance with the National Yang-Ming University Institutional Animal Care and Use Committee (IACUC). The 4-nitroquinoline-1-oxide (4NQO, Sigma) solution was made freshly by dissolved in 1,2-propylene glycol (Sigma) and added directly to water to a final concentration of 100 μg/ml. Water was changed weekly. For STZ-induced DM, daily injection of 50mg/kg STZ was given in 6–8 weeks old male C57/BL6 intraperitoneally for 5 consecutive days. Control mice were injected with buffer alone. Blood glucose levels were followed after the final injection weekly using a glucometer (Optium Xceed, Abbott). Mice with blood glucose levels greater than 350 mg/dl were considered hyperglycemia.

### Immunohistochemistry

Cells were seeded on 20 × 20 mm cover slides and cultured in indicated medium. The cells cultured on cover slides were fixed with 4% PFA for 20–30 minutes and incubated with PBS in 4°C until use. The primary and secondary antibodies were sequentially applied onto the cells and DAPI was used as counter stain to define cell nuclei. Stainings were examined using Leica DM 6000B fluorescence microscope or Olympus FV1000 Confocal microscope at Instrumentation Resource Center, National Yang-Ming University. Final images were processed using Adobe Photoshop. For F-actin staining, cells were treated and fixed as previous Immunohistochemistry assay. Cellular F-actin staining has been developed as alternative fluorescent marker for actin cytoskeleton in living cells. The primary components of staining was phalloidin, which was purified from a mixture of mushroom toxins residual. F-actin staining was applied on cells than follow with DAPI staining. For quantification of 4-NQO induced tumor lesions, the H&E staining for at least 3 individual tongue tissue sections apart from each other by at least 50microns from same animal were examined to determine the tumor index. All stainings were examined using Leica DM 6000B fluorescence microscope or Olympus FV1000 Confocal microscope at Instrumentation Resource Center, National Yang-Ming University. Final images were processed using Adobe Photoshop.

### Clinical information

A consecutive cohort of 869 patients from January 1^st^, 2002 to December 31^st^, 2007 was retrieved from Taipei MacKay Memorial Hospital. Patients were enrolled for the study when the following inclusion criteria were met; (1) histologically diagnosed as squamous cell carcinoma and (2) received definitive surgical intervention as the initial treatment modality. The exclusion criteria were: (1) recurrent or metastatic disease, (2) previously treated with radiation or chemotherapy, and (3) history of synchronous or metachronous cancers. Based on these criteria, 613 patients were enrolled for subsequent analysis. Tumor staging was defined according to the American Joint Committee on Cancer (AJCC 7th) system of tumor, node, metastasis (TNM) classification. Patients with positive cervical lymph nodes, perineural invasion, perivascular or perilymphangenic invasion, nodal extracapsule spread and closed margin had received post-operative adjuvant concurrent chemo/radiation treatment according to the National Comprehensive Cancer Network (NCCN) guidelines. DM was determined by a fasting plasma glucose (FPG) level over 126 mg/dL, a non-FPG level over 200 mg/dl or an pre-operative HbA1c level over 6.5%. Patients received medications for diabetes and those with a history of such drugs therapy were also evaluated as having DM. We divided the oral cancer patients into 2 groups: patients with DM (DM group) and those without DM (non-DM group). Patients were followed for an average period of 54.5 months.

### Statistical analysis

All analyses were performed using statistical software program package Prism 5 (GraphPad, San Diego, CA) and SPSS 18.0 (SPSS Inc, Chicago, IL). The differences in the clinical characteristics between two groups were analyzed by a Chi-square test and student's *t* test. Overall survival (OS) was calculated from the day of definite surgery to the date of death from any cause or the date of the last follow-up. Disease free survival (DFS) was defined as the time from the day of surgery till progression or death. OS and DFS were estimated using the Kaplan-Meier method. The Cox proportional hazards regression model was used to determine statistical significant variables related to survival. Differences were assumed to be significant when *p*-value < 0.05.

## SUPPLEMENTARY MATERIAL FIGURES AND TABLES


