# Supplementary material for: Dynamic cellular and molecular modulations of diabetes mediated head and neck carcinogenesis
Source: Oncotarget. 2015 Aug 21;6(30):29268–84. doi: 10.18632/oncotarget.4922 (PMC4745725; doi:10.18632/oncotarget.4922)
Supplement: Supplementary file 1 [file oncotarget-06-29268-s001.pdf]

SUPPLEMENTARY FIGURES AND TABLES

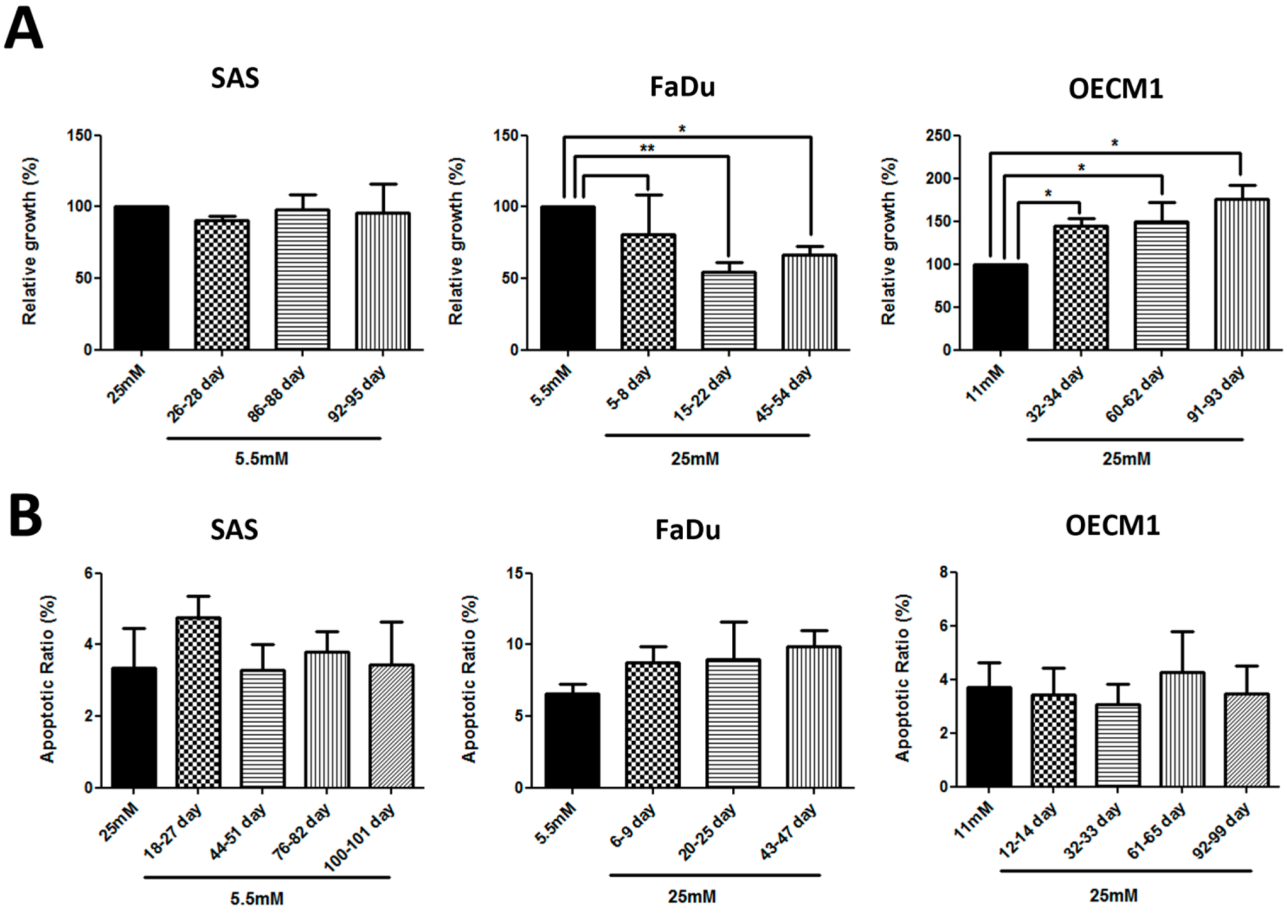

Supplementary Figure S1: A. Cell growth by trypanblue exclusion assay and **B.** apoptotic index using FITC AnnexinV Apoptosis Kit for head and neck caner cells cultured in low and high glucose medium. ***p* < 0.01; **p* < 0.05; n.s. = non significant.

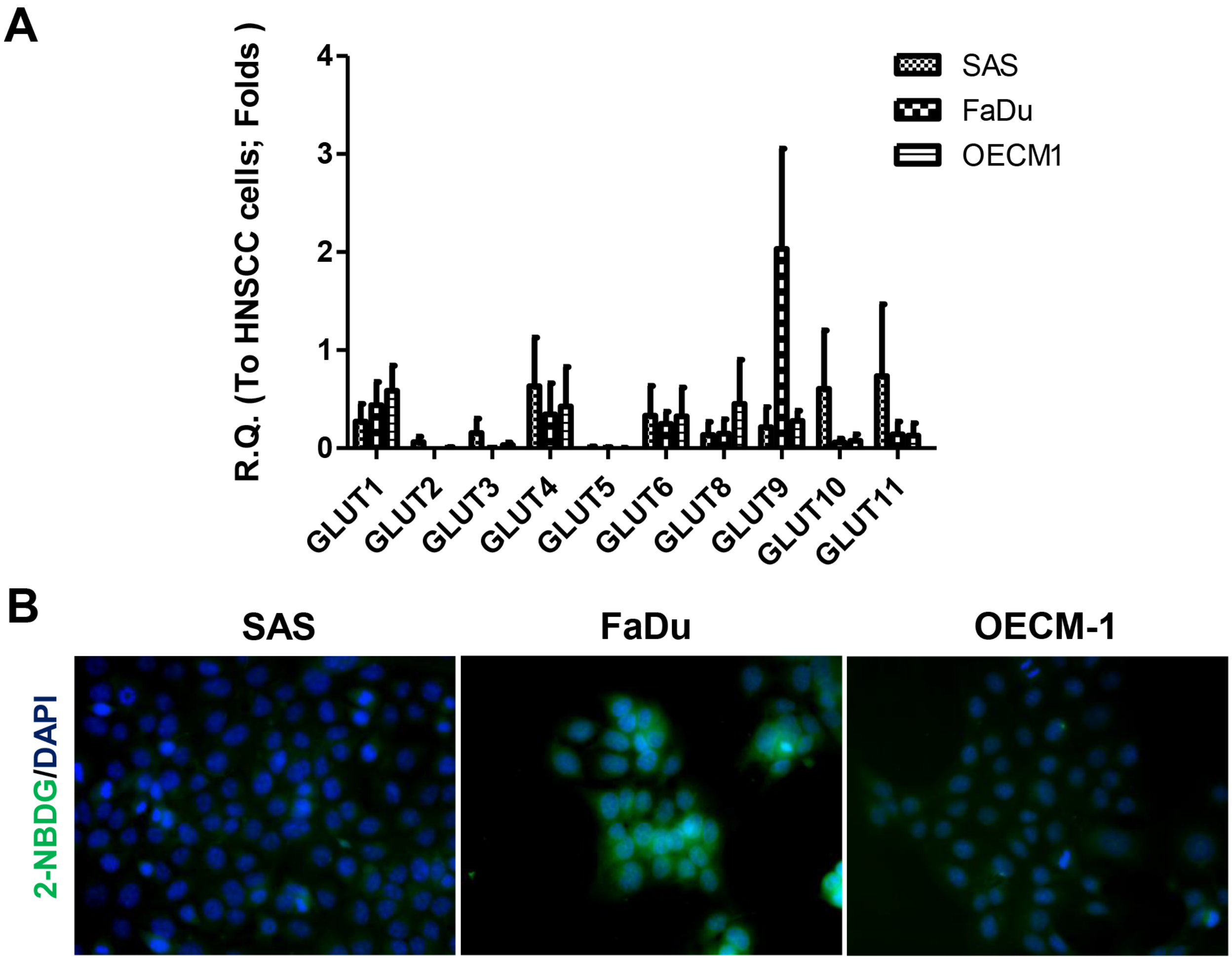

Supplementary Figure S2: **A.** real-time RT-PCR analysis and **B.** 2-NBDG uptake assay for glucose transporter (Gluts) mRNA expression showed differential glucose uptake activity among SAS, FaDu and OECM-1 cells.

A

SAS

Treatment	BAAA	BAAA + DEAB
25mM	0.16 ±0.005%	0.12 %
5.5mM, 27d	0.40±0.175 %	0.11 %
5.5mM, 52d	0.34±0.54 %	0.11 %
5.5mM, 82d	0.26 ±0.12%	0.11 %
5.5mM, 105d	0.19±0.655 %	0.10 %

FaDu

Treatment	BAAA	BAAA + DEAB
5.5mM	0.56± 0.005%	0.12 %
25mM, 7-8d	0.48±0.02 %	0.12 %
25mM, 24-37d	0.67±0.19 %	0.14 %
25mM, 45-46d	1.025±0.155 %	0.12 %

OECM1

Treatment	BAAA	BAAA + DEAB
11mM	3± 1.7%	0.10 %
25mM, 12d	2.7±0.7 %	0.11 %
25mM, 27-34d	3.5±1.0 %	0.11 %
25mM, 56-62d	2.39±0.23 %	0.11 %
25mM, 93-100d	2.8±1.01 %	0.11 %

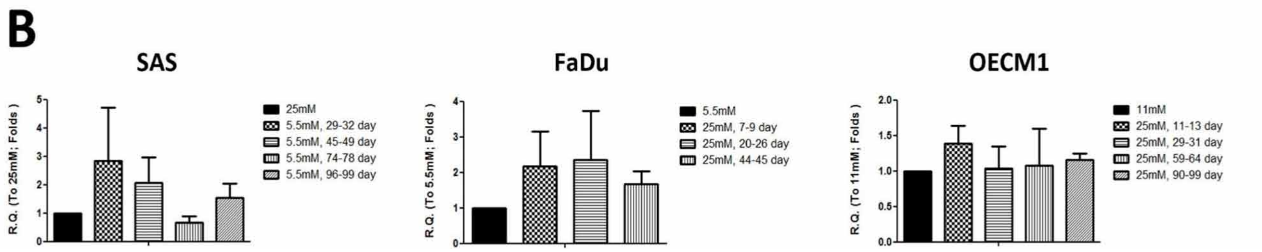

Supplementary Figure S3: No significant difference was detected for **A.** ALDH activity and **B.** mRNA expression of stemnessmarker Oct4 in head and neck cancer cells cultured in low and high glucose.

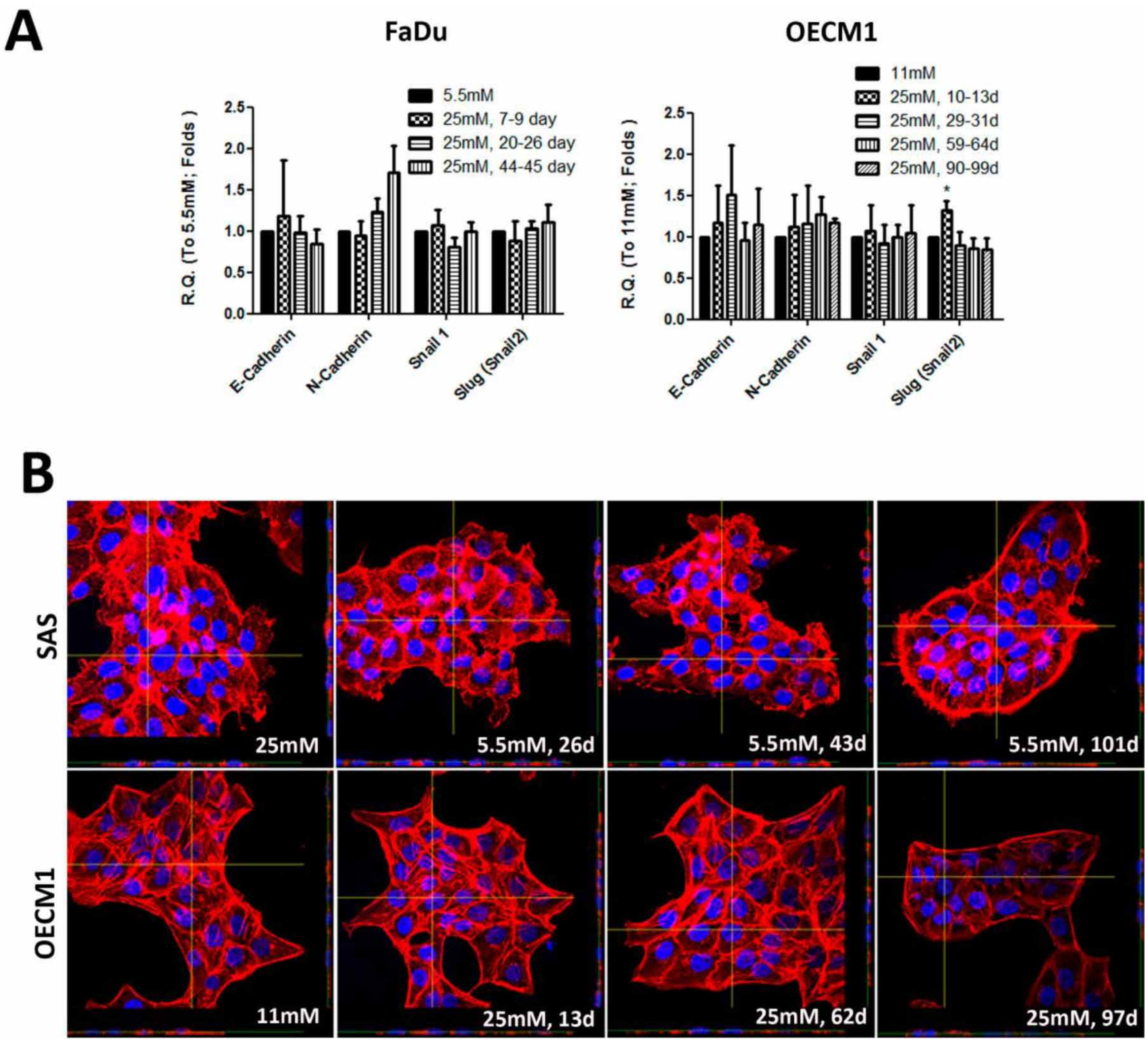

Supplementary Figure S4: A. No significant change EMT mRNA in Faduand OECM1 cells cultured with medium containing differential glucose levels over time. **B.** Z-stack profile of F-actin in SAS and OCEM1 cell in medium with differential glucose concentrations

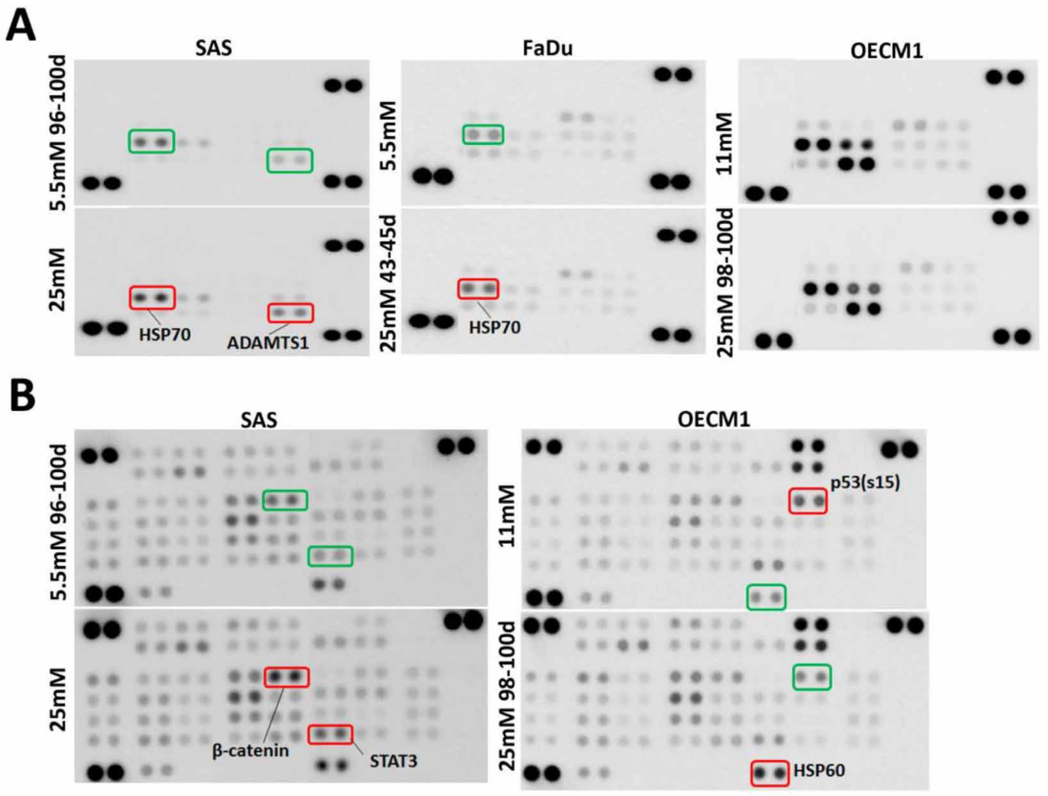

C

Gene Changed in High Glucose (25mM)	Up-regulated	Down-regulated
SAS	HSP70	--
	ADAMTS1	
	STAT3 (Y705)	
	β-catenin (-)	
FaDu	HSP70	--
	PRAS40 (T246)	
	WNK1 (T60)	
OECM1	HSP60 (-)	p53 (S15)

Supplementary Figure S5: A. Human cell stress and **B.** Human phospho-kinase antibody arrays for head and neck caner cells cultured in low and high glucose medium. Green and red boxes highlighted down-regulated and up-regulated proteins, respectively. The result is summarized in **C.**

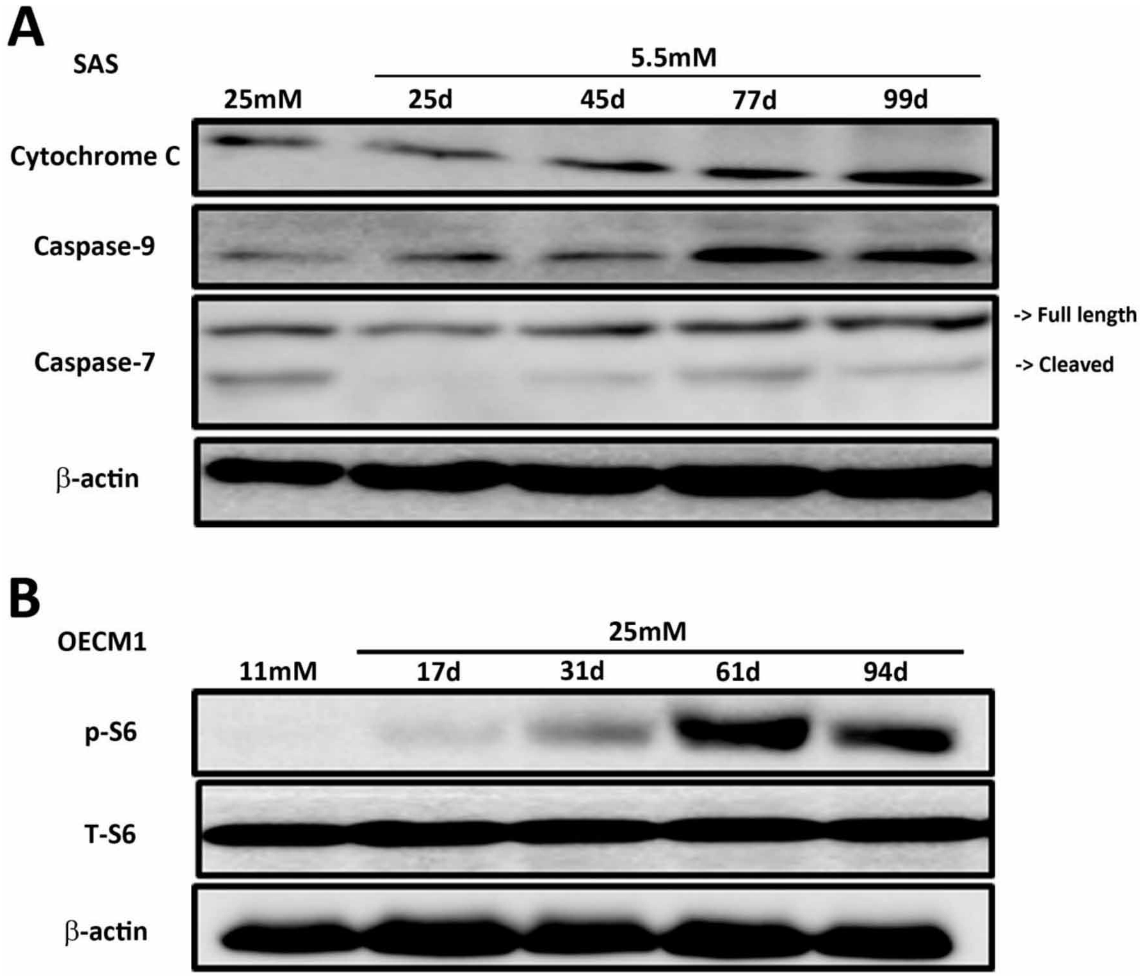

Supplementary Figure S6: A. Up-regulated Cytochrome c mediated apoptotic pathway in SAS cells treated with long-term high-glucose; **B.** Expression of mTORCdownstream target phospho-S6 protein is increased in OECM-1 treated with high glucose.

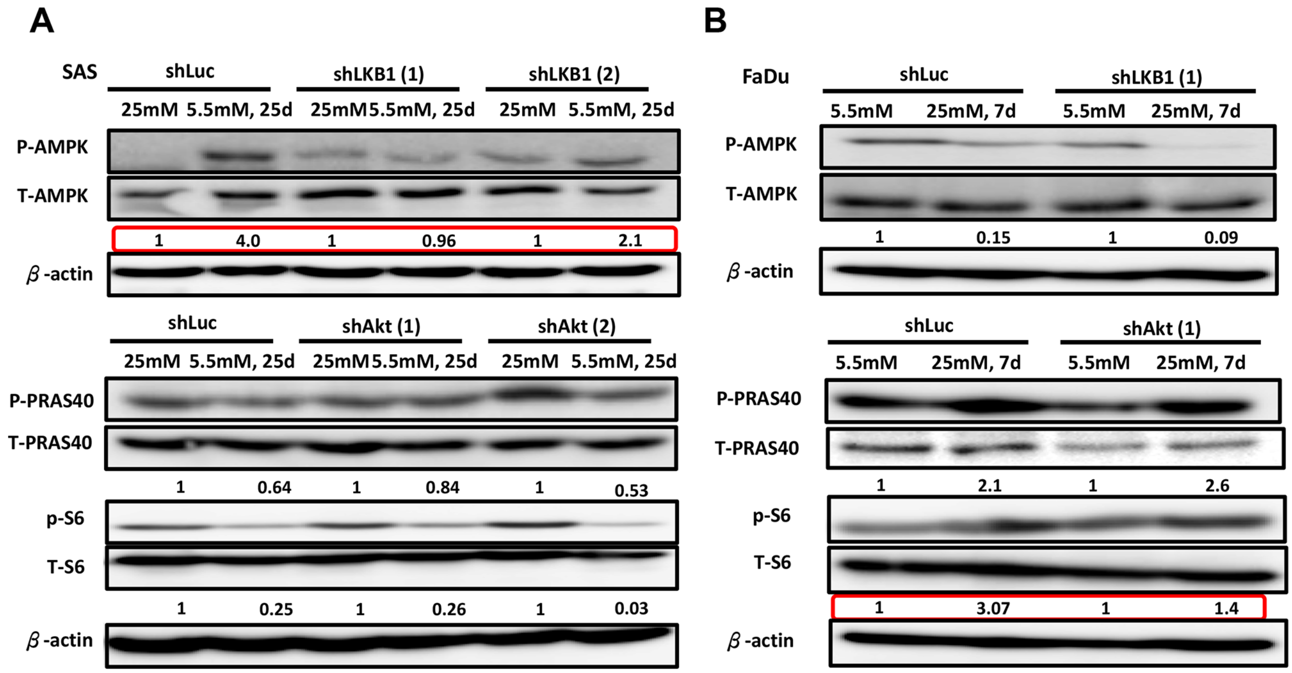

Supplementary Figure S7: Molecular modulation in Akt and LKB1 deficient SAS and FaDu cells under differential glycemic cultures. The LKB1 downstream AMPK activity is abolished in Akt deficient SAS cells cultured in low-glucose medium whereas activation of S6 protein is blocked in FaDu cells cultivated in medium containing 25 mM glucose (highlighted in red box).

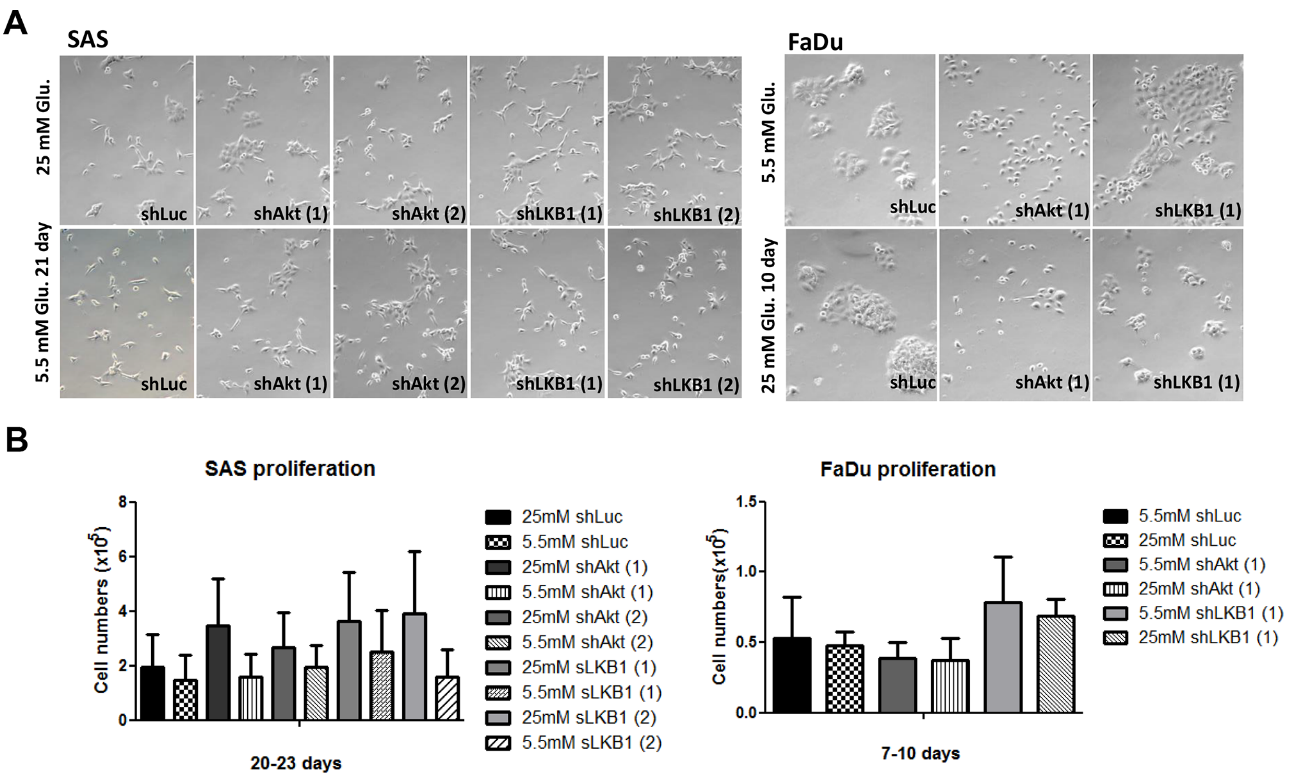

Supplementary Figure S8: No significant changes of **A.** cell morphology and **B.** cell proliferation (Trypanblue exclusion assay) in Aktor LKB1 deficient SAS/FaDu cells compared with shLucinfected control cells.

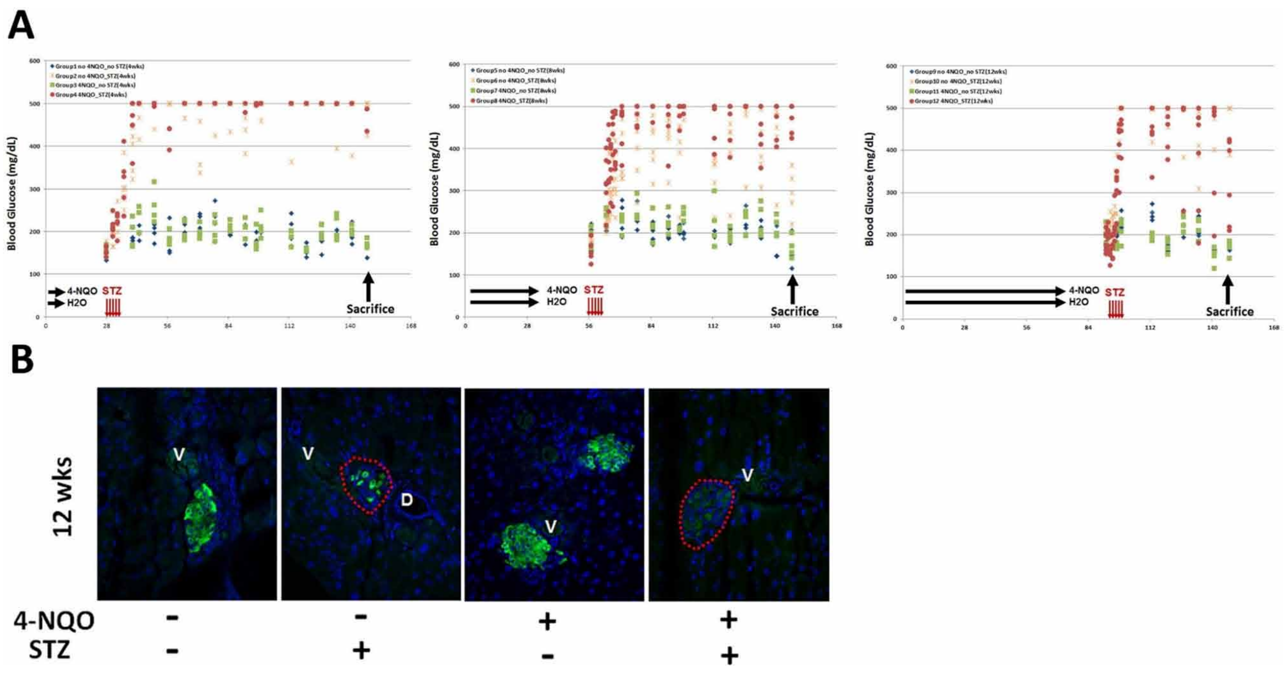

Supplementary Figure S9: A. Blood glucose levels remain high until sacrifice in STZ-injected mice with or without treatment of oral carcinogen 4-NQO; **B.** Insulin-producing cells (green) in islets (red lined) disrupted upon treatment of STZ. V: Vessel, D: Ducts.

Supplementary Table S1: Primers, antibodies and shRNA plasmid sequences used in present study

A.

Primary antibody	Species	Vendor	Application
Anti-involucrin	Mouse	Sigma	IHC/WB
Anti-cytochrome C	Mouse	Abcam	WB
Anti-caspase-9	Mouse	R&D	WB
Anti-caspase-3	Rabbit	Cell signaling	WB
Anti-caspase-7	Mouse	R&D	WB
Anti-Akt	Rabbit	Cell signaling	WB
Anti-p-Akt	Rabbit	Cell signaling	WB
Anti-PRAS40	Mouse	R&D	WB
Anti-p-PRAS40	Mouse	R&D	WB
Anti-LKB1	Rabbit	Cell signaling	WB
Anti-p-LKB1	Rabbit	Cell signaling	WB
Anti-AMPKα	Rabbit	Cell signaling	WB
Anti-p-AMPKα (Thr 172)	Rabbit	Cell signaling	WB
41701.00	Rabbit	Cell signaling	WB
Anti-Raptor	Rabbit	Cell signaling	WB
Anti-p-Raptor	Rabbit	Cell signaling	WB
Anti-S6k	Rabbit	Cell signaling	WB
Anti-p-S6k	Rabbit	Cell signaling	WB
Anti-S6	Rabbit	Cell signaling	WB
Anti-p-S6 (Ser 235/236)	Rabbit	Cell signaling	WB

(*Continued*)

Primary antibody	Species	Vendor	Application
E-Cadherin	Rabbit	Abcam	WB
N-Cadherin	Mouse	BD Biosciences	WB
Snail (L70G2)	Mouse	Cell signaling	WB
Slug	Rabbit	Abcam	WB
Anti-β-actin	Mouse	Novus Biological	WB
Anti-E-Cadherin	Mouse	Abcam	IF
Anti-Ki-67	Rabbit	Abcam	IHC
Anti-Insulin	Guinea pig	Abcam	IF

B.

Secondary antibody	Species	Vendor	Application
Anti-mouse HRP	Donkey	Jackson Immuno Research	WB
Anti-rabbit HRP	Donkey	Jackson Immuno Research	WB
Biotinylated anti-goat	Rabbit	Jackson Immuno Research	IHC
Alexa Fluor 488 Anti-mouse	Donkey	Jackson Immuno Research	IF
Alexa Fluor 594 Anti-mouse	Donkey	Jackson Immuno Research	IF
Alexa Fluor 647 Anti-guinea pig	Goat	Abcam	IF

C.

Human E-Cadherin	
Forward	5'-GACTCGTAACGACGTTGCAC-3'
Reverse	5'-GGTCAGTATCAGCCGCTTTC-3'
**Human N-Cadherin**	
Forward	5'-CTGGAGACATTGGGGACTTC-3'
Reverse	5'-CATAGTCCTGCTCACCACCA-3'
**Human Snail**	
Forward	5'-GAAAGGCCTTCAACTGCAAA-3'
Reverse	5'-CAGCCAGGGCCTAGAGAAG-3'
**Human Slug (Snail 2)**	
Forward	5'-GAGCATTTGCAGACAGGTCA-3'
Reverse	5'-CCTCATGTTTGTGCAGGAGA-3'

D.

Gene	Region	Clone ID	Target Sequence
**Akt1**	CDS	TRCN0000010162	GGACAAGGACGGGCACATTAA
	CDS	TRCN0000010163	CGAGTTTGAGTACCTGAAGCT
**STK11 (LKB1)**	CDS	TRCN0000000408	GCCAACGTGAAGAAGGAAATT
	CDS	TRCN0000000410	GAAGAAGAAGTTGCGAAGGAT

Supplementary Table S2: Clinopathological parameters of patients with or without DM included in current study

	Non-DM (*N* = 487)	DM (*N* = 126)	Total patients (*N* = 613)
**Gender**			
Male	454	115	569
Female	33	11	44
**Tobacco smoking**			
Daily	290	70	360
Non-smoker	197	56	253
**Alcohol abuse**			
Daily	51	15	66
None or social	436	111	547
**Age** (average year)	52.46	56.42	53.24
**Site**			
Buccal mucosa	181	50	231
Tongue	127	43	170
Gingiva	104	20	124
Oral pharynx	21	4	25
Hypopharynx	5	2	7
Other	52	4	56
**Tumor size**			
T1–T3	215	45	260
T4	272	81	353
***N* status**			
N = 0	319	55	374
N > 0	168	71	239
**Tumor stage**			
I–II	126	14	126
III–IV	361	112	487
**Lymphovascular permeation**			
Negative	497	92	489
Positive	80	34	114
**Cell differentation**			
Well	178	42	220
Moderate	278	73	351
Poor	31	11	42

(*Continued*)

	Non-DM ($N = 487$)	DM ($N = 126$)	Total patients ($N = 613$)
**Perineural invasion**			
Negative	385	95	480
Positive	102	31	133
**Follow-up duration** (median month)	53.1	52.3	52.9
**Survival status**			
Dead	128	70	198
Survived	359	56	415

Supplementary Table S3: Association between clinicopathological parameters and survival of HNSCC patients

	Total		Dead		Survived		*P*-value	OR	95% C.I.	*P*-value
**Gender**							0.351			
Male	569	92.80%	181	91.40%	388	93.50%				
Female	44	7.20%	17	8.60%	27	6.50%				
**Age** (yr)	53.28±11.06		54.17±11.39		52.85±10.89		0.169			
***N* status**							$p < 0.001$			
$N = 0$	372	60.90%	78	39.40%	294	71.20%		1.00		
$N > 0$	239	39.10%	120	60.60%	119	28.80%		2.13	1.34–3.39	0.001
**DM**							$p < 0.001$			
yes	126	20.60%	70	35.40%	56	13.50%		2.89	1.84–4.54	$p < 0.001$
no	487	79.40%	128	64.60%	359	86.50%		1.00		
**Tumor size**							$p < 0.001$			
T1–T3	260	42.60%	44	22.20%	216	52.30%		1.00		
T4	351	57.40%	154	77.80%	197	47.70%		2.91	1.73–4.9	$p < 0.001$
**Stage**							$p < 0.001$			
I–II	140	22.90%	12	6.10%	128	31.00%		1.00		
III–IV	471	77.10%	186	93.90%	285	69.00%		1.51	0.67–3.42	0.318
**Lymphovascular permeation**							$p < 0.001$			
Positive	489	81.40%	133	68.60%	356	87.50%		1.49	0.88–2.52	0.142
Negative	112	18.60%	61	31.40%	51	12.50%		1.00		
**Cell differentation**							$p < 0.001$			
Well	220	35.90%	50	25.30%	170	41.00%		1.00		
Moderate	351	57.30%	126	63.60%	225	54.20%		1.73	1.12–2.67	0.014
Poor	42	6.90%	22	11.10%	20	4.80%		2.44	1.12–5.32	0.025

(*Continued*)

	**Total**		**Dead**		**Survived**		***P*-value**	**OR**	**95% C.I.**	***P*-value**
**Perineural invasion**							$p < 0.001$			
Negative	472	78.00%	130	67.00%	342	83.20%		1.00		
Positive	133	22.00%	64	33.00%	69	16.80%		1.28	0.8–2.05	0.307

Multivariate logistical regression analysis showed lymph node, tumor size, cell differentiation and the DM status were associated with survival.
